# Evaluation of a multiplex-qPCR for paediatric pleural empyema—An observational study in hospitalised children

**DOI:** 10.1371/journal.pone.0304861

**Published:** 2024-06-25

**Authors:** Jonathan Jacobson, Loraine Fabri, Joshua Osowicki, Shivanthan Shanthikumar, Anna-Maria Costa, Belinda Ortika, Ashleigh Wee-Hee, Michelle Pragassen, Cassandra Gatt, Gena Gonis, Cattram Nguyen, Thomas Rozen, Warwick Teague, Jim Buttery, Vanessa Clifford, Kim Mulholland, Andrew Steer, Sarath Ranganathan, Andrew Daley, Eileen Dunne, Catherine Satzke

**Affiliations:** 1 Infection, Immunity and Global Health, Murdoch Children’s Research Institute, Royal Children’s Hospital, Parkville, Victoria, Australia; 2 Paediatric Department, Academic Children Hospital Queen Fabiola, Université Libre de Bruxelles, Brussels, Belgium; 3 Department of Paediatrics, University of Melbourne, Parkville, Victoria, Australia; 4 Infectious Diseases Unit, Department of General Medicine, The Royal Children’s Hospital Parkville, Parkville, Victoria, Australia; 5 Respiratory and Sleep Medicine, The Royal Children’s Hospital, Parkville, Victoria, Australia; 6 Department of Microbiology, The Royal Children’s Hospital, Parkville, Victoria, Australia; 7 Complex Care Hub, The Royal Children’s Hospital, Parkville, Victoria, Australia; 8 Clinical and Epidemiology Biostatistics Unit, Murdoch Children’s Research Institute and The Royal Children’s Hospital, Parkville, Victoria, Australia; 9 Department of Paediatric Surgery, The Royal Children’s Hospital, Parkville, Victoria, Australia; 10 Surgical Research Group, Murdoch Children’s Research Institute, Parkville, Victoria, Australia; 11 Infection and Immunity, Monash Children’s Hospital, Clayton, Victoria, Australia; 12 Department of Paediatrics & Monash Centre for Health Care Research and Implementation, Monash University, Clayton, Victoria, Australia; 13 Department of Medicine, Dentistry and Health Sciences, University of Melbourne, Parkville, Victoria, Australia; 14 Department of Microbiology and Immunology, The Peter Doherty Institute for Infection and Immunity, Parkville, Victoria, Australia; Universidade de Lisboa Faculdade de Medicina, PORTUGAL

## Abstract

Pleural empyema is a serious complication of pneumonia in children. Negative bacterial cultures commonly impede optimal antibiotic therapy. To improve bacterial identification, we developed a molecular assay and evaluated its performance compared with bacterial culture. Our multiplex-quantitative PCR to detect *Streptococcus pneumoniae*, *Streptococcus pyogenes*, *Staphylococcus aureus* and *Haemophilus influenzae* was assessed using bacterial genomic DNA and laboratory-prepared samples (n = 267). To evaluate clinical performance, we conducted the Molecular Assessment of Thoracic Empyema (MATE) observational study, enrolling children hospitalised with empyema. Pleural fluids were tested by bacterial culture and multiplex-qPCR, and performance determined using a study gold standard. We determined clinical sensitivity and time-to-organism-identification to assess the potential of the multiplex-qPCR to reduce the duration of empiric untargeted antibiotic therapy. Using spiked samples, the multiplex-qPCR demonstrated 213/215 (99.1%) sensitivity and 52/52 (100%) specificity for all organisms. During May 2019–March 2023, 100 children were enrolled in the MATE study; median age was 3.9 years (IQR 2–5.6). A bacterial pathogen was identified in 90/100 (90%) specimens by multiplex-qPCR, and 24/100 (24%) by bacterial culture (*P* <0.001). Multiplex-qPCR identified a bacterial cause in 68/76 (90%) culture-negative specimens. *S*. *pneumoniae* was the most common pathogen, identified in 67/100 (67%) specimens. We estimate our multiplex-qPCR would have reduced the duration of untargeted antibiotic therapy in 61% of cases by a median 20 days (IQR 17.5–23, range 1–55). Multiplex-qPCR significantly increased pathogen detection compared with culture and may allow for reducing the duration of untargeted antibiotic therapy.

## Introduction

Pleural empyema (hereafter ‘empyema’) describes a collection of pus in the pleural cavity, and is a well-recognized complication of community acquired pneumonia (CAP). Empyema occurs in approximately 1% of paediatric CAP cases in Australia [1, 2]. While paediatric admissions for CAP have reduced following the introduction of *Haemophilus influenzae* type b and pneumococcal conjugate vaccines (PCVs), rates of empyema have increased within the same timeframe [1]. The most common bacterial causes are *Streptococcus pneumoniae*, *Streptococcus pyogenes*, *Staphylococcus aureus* and *Haemophilus influenzae* [3].

Children with empyema are initially treated with empiric, broad-spectrum (untargeted) antibiotic therapy, covering the most relevant bacterial pathogens [4]. Routine clinical care for empyema includes drainage of pleural fluid, which can be tested to identify the causative bacterial species [5]. Identifying the bacterial etiology allows for transition to targeted antibiotic therapy, minimizing duration of untargeted antibiotic treatment. This reduces the risk of antibiotic-related side effects and antibiotic resistance arising [6]. Bacterial culture alone takes one to five days to isolate and identify a causative organism [7], and, due to antibiotic therapy preceding drainage, frequently fails to identify an organism, even with optimum enrichment techniques [8]. Without a confirmed bacterial cause, untargeted antibiotics are typically continued for the entire course of treatment, which is often longer than 4 weeks in duration [4, 9].

Molecular methods are increasingly applied across clinical microbiology, particularly for specimens and pathogens, where culture-based methods are unlikely to identify the cause. Species-specific quantitative PCR (qPCR) assays for detection of *S*. *pneumoniae*, *S*. *pyogenes*, *S*. *aureus*, and *H*. *influenzae* have been developed and applied to a range of clinical sample types [10–13]. In this study, we aimed to develop a multiplex-qPCR for empyema, evaluate its performance compared with culture, and consider its potential clinical impact.

## Materials and methods

### Multiplex-qPCR assay

Primers and probes for *S*. *pneumoniae*, *S*. *pyogenes*, *H*. *influenzae* and *S*. *aureus* have been previously described (S1 Table). Each 25 μl reaction included: 1x Brilliant III Ultra-Fast qPCR master mix (Agilent), primers at 100 nM (Sigma-Aldrich), probes at 250 nM (Integrated Sciences) and 5 μl purified bacterial genomic DNA. Assays were performed using an AriaMx Real-Time PCR system (Agilent). Cycling conditions were: 3 min at 95°C, with 45 amplification cycles of 95°C for 20 s and 58°C for 40 s. Cq values were generated using AriaMx software v.1.71 (Agilent). Samples with a Cq value ≤ 35 were considered positive and > 35 were considered negative [11, 12]. Results are presented in accordance with the MIQE guidelines for qPCR assays [14].

### Performance of the multiplex-qPCR

i) To determine the limits of detection, bacterial genomic DNA was extracted as previously described [15] and diluted in a 10-fold series from 5x10^6^ genome equivalents (GE) to 5 GE in nuclease-free water before assaying. ii) To rigorously assess our multiplex-qPCR assay, thirteen bacterial isolates (S2 Table) were used to create 267 laboratory-prepared (spiked) samples (S3 Table). Isolates were grown overnight at 37°C and 5% CO_2_ in 5 ml of THY media: Todd Hewitt Broth (Sigma-Aldrich) with 0.5% (w/v) yeast extract (Sigma-Aldrich). Aliquots (1 ml) of sterile pleural fluid (MyBioSource) were spiked with one (n = 154) or multiple (n = 61) bacterial species, to concentrations ranging between 1.0 x 10^4^ cfu/ml to 1.0 x 10^8^ cfu/ml, or with sterile media (no bacteria, n = 52). Spiked samples were immediately placed at -80°C for >24 h before DNA extraction and testing by multiplex-qPCR.

### DNA extraction from pleural fluid

Pleural fluid samples (200 μl) were centrifuged at 10,000 x *g*. Pellets were resuspended in an enzymatic digestion buffer containing 5 mM EDTA (Sigma Aldrich), 3 mg/ml lysozyme (Sigma Aldrich), 37.5 μg/ml mutanolysin (Sigma Aldrich), 150 μg/ml lysostaphin (Sigma Aldrich), 1% Triton X 100 (Sigma Aldrich) and 2 mg/ml RNAse A (QIAgen). Following incubation for 1 h at 37°C, 180 μl of buffer ATL (QIAamp DNA mini kit, Qiagen) was added with Proteinase K to 1 mg/ml. Following another incubation step (30 min at 56°C), 200 μl of lysis buffer AL (QIAamp DNA mini kit, Qiagen) was added, and tubes were incubated for 10 min at 70°C. DNA was isolated using the QIAcube HT platform (Qiagen) according to the manufacturer’s instructions and eluted in 100 μl of elution buffer.

### Observational study and bacterial identification using pleural fluid specimens

From 6^th^ May 2019 to 1^st^ March 2023, participants were enrolled into the Molecular Assessment of Thoracic Empyema (MATE) study at the Royal Children’s Hospital Melbourne (RCH), the largest tertiary paediatric hospital in the State of Victoria, Australia. Patients <18 years of age and hospitalized with CAP complicated with diagnosed empyema and who had pleural fluid drained were eligible for enrolment following parent/guardian written consent. Where consent was given verbally by telephone, consent forms were signed and dated by a team member. Patients were excluded if consent was not given. Eligible patients were identified by searching the electronic medical record search for ICD codes relating to infectious pneumonia and empyema. There were n = 105 eligible patients identified throughout the project term, of which n = 100 were enrolled. Three participants were not enrolled as we were unable to contact them for consent. Two patient’s families did not wish to participate in the MATE study. Patients with empyema from non-infectious causes were ineligible and were not approached. Electronic medical records were examined for relevant baseline and clinical information including: vaccination status, admissions to the Intensive Care Unit (ICU) and duration of antibiotic usage. Pleural fluid specimens were collected during the routine clinical care by percutaneous chest tube insertion or during Video assisted thoracoscopic surgery (VATS). Bacterial culture performed by the RCH’s Department of Microbiology. For bacterial culture, pleural fluid samples were streaked for single colonies onto MacConkey-, chocolate- and blood-agar plates, as well as being inoculated into cooked meat media broth with incubation up to five days. Isolates were tested to identify the bacterial species following relevant protocols in the Clinical Microbiology Procedures Handbook [16]. Remaining pleural fluid was retained at 4°C for the MATE study and stored at -80°C within one week of drainage. DNA was extracted from pleural fluids and multiplex-qPCR assays performed. The reporting of this study conforms to the STROBE statement [17]. As this study was conducted as an evaluation, multiplex-qPCR was conducted in batches and results were not reported back to clinicians involved in patient care.

### Assessment of potential clinical benefit

The potential impact of the multiplex-qPCR on antibiotic prescribing was estimated. For each participant, we recorded the duration of empiric untargeted antibiotic treatment and whether a species was identified by standard bacterial culture. For culture-positive pleural fluid specimens, we also obtained the time-to-result. We then conservatively assumed that the multiplex-qPCR would achieve a 48-hour turnaround time, if integrated into existing workflows, to calculate the potential reduction in the duration of empiric untargeted antibiotic therapy. For example, if a participant had no organism identified by culture and received 14 days of empiric antibiotic therapy after pleural drainage, and the multiplex-qPCR was positive, there would be a 12-day reduction in the duration of empiric untargeted antibiotic therapy.

### Statistical analysis

Descriptive statistics were expressed as a percentage, or median with interquartile range (IQR) or range where stated. Sensitivity, specificity, positive predictive value, and negative predictive value were calculated for the multiplex-qPCR by examining spiked samples. The performance of multiplex-qPCR and bacterial culture were compared against a composite study gold standard, defined as a positive result by either method. Detection of bacterial pathogens in clinical specimens by the two methods was compared using M^c^Nemar’s χ^2^ test and two-tailed p values reported. Statistical analyses were conducted using Prism 7.0d (GraphPad Software).

### Ethics

Ethical approval was obtained from the Royal Children’s Hospital (HREC 38369). Consent was given by parents/guardians and permission was granted by the Australian Immunisation Register to access enrolled participants’ immunisation histories. Records were accessed on 10^th^ October 2023.

## Results

### Multiplex qPCR performance using laboratory prepared samples

Using purified bacterial genomic DNA, we determined the limit of detection for each of the target gene ranged between 5–50 GE (S1 Fig). The multiplex-qPCR returned the correct result for 265/267 spiked samples that contained either zero (n = 52) or between one and four bacterial species (n = 215), equating to 99.1% sensitivity and 100% specificity (Table 1). False negative results were returned for two spiked samples: one containing *S*. *pyogenes* and another containing *S*. *pyogenes* and *S*. *aureus*. There were no false positive results.

**Table 1:** Performance of multiplex-qPCR when testing spiked samples.

	Sensitivity	Specificity
**All species**	213/215 (99.1)	52/52 (100)
***S*. *pneumoniae***	108/108 (100)	159/159 (100)
***S*. *pyogenes***	74/76 (97.4)	191/191 (100)
***H*. *influenzae***	57/57 (100)	210/210 (100)
***S*. *aureus***	66/67 (98.5)	200/200 (100)

Sensitivity calculated as no. true positive / no. known positive (%); Specificity calculated as no. true negative / no. known negative (%).

### MATE study participant characteristics

One hundred children were enrolled in the MATE study (Table 2). The median age of participants was 3.9 years (IQR 2–5.6 years). At admission, 90 (90%) children had received the appropriate number of 13-valent PCV (13vPCV) doses for their age. Doses of 13vPCV were administered as a 3+0 schedule (at 2, 4 and 6 months of age), or a 2+1 schedule (2 and 4 months, with a booster at 12 months of age), as recommended by the Australian National Immunisation Program. Forty-one (41%) children were admitted to the ICU. Non-invasive respiratory support (low or high flow nasal canula, bilevel or continuous positive airway pressure) was required in 44 (44%) cases, with 10 (10%) patients requiring additional invasive ventilation. VATS was the primary surgical intervention in 70/100 (70%) cases. For all enrolled children, antibiotic therapy commenced either prior to, or on the day of primary pleural fluid drainage. The median number of different antibiotics administered throughout treatment was 5 (IQR 4–6) for a median total duration of 25 days (IQR 22–31). The median duration of hospital stay was 11 days (IQR 8–15).

**Table 2:** Characteristics of study participants.

Characteristic	All patients n = 100
**Age, median (IQR), years**	3.9 (2–5.6)
**Male, n (%)**	53 (53)
**Fully PCV immunised** ^**a**^ **, n (%)**	90 (90)
**Aboriginal and/or Torres Strait Islander, n (%)**	4 (4)
**Clinical presentation at admission, n (%)**	
Fever (≥ 38°C)	95 (95)
Hypoxia (oxygen saturation level ≤ 92%)	73 (73)
X-ray performed	100 (100)
Ultrasound performed	97 (97)
**ICU admission, n (%)**	41(41)
**Respiratory support, n (%)**	
Non-invasive^b^	44 (44)
Invasive ventilation	10 (10)
**Duration respiratory support, median (IQR), days**	5 (3–8)
**Primary intervention, n (%)**	
Video assisted thoracoscopic surgery	70 (70)
Chest drainage and fibrinolytic therapy	29 (29)
Sternotomy	1 (1)
**Required reintervention, n (%)**	12 (12)
**Antibiotics administered prior to primary intervention, n (%)**	100 (100)
**Antibiotics administered (number of different antibiotics)**	
Median (IQR)	5 (4–6)
**Duration of antibiotic therapy, median (IQR), days**	
Intravenous	10 (7–14)
Oral	15 (14–16)
Total	25 (22–31)
**Duration of hospital admission, median (IQR), days**	11 (8–15)
**Clinical outcome, n (%)**	
Recovery and discharge	99 (99)
Death	1 (1)

Abbreviations: IQR, interquartile range. PCV, pneumococcal conjugate vaccine. ICU, intensive care unit

^a^ Children had received the appropriate number of pneumococcal conjugate vaccine (PCV) doses for their age as recommended by the Australian National Immunisation Program

^b^ Low/High flow nasal cannula, bilevel/continuous positive airway pressure (BiPAP/CPAP)

### Bacterial etiology of empyema

Pleural fluid specimens (one per child) were collected and assessed. A bacterial species was identified in 24/100 (24%) specimens by bacterial culture, 90/100 (90%) by multiplex-qPCR, McNemar’s χ^2^ 62.2, p<0.0001 and in 92/100 (92%) by both methods together (Table 3). *S*. *pneumoniae* was the most common pathogen, found in 67/100 (67%) pleural fluids. *S*. *pyogenes* was the next most common, in 21/100 (21%), followed by *H*. *influenzae* 2/100 (2%) and *S*. *aureus* 2/100 (2%) (S2 Fig). For 22/24 (92%) culture-positive specimens, the multiplex-qPCR obtained the same result. In two culture-positive specimens, the multiplex-qPCR failed to detect *S*. *pneumoniae* for one specimen and *S*. *pyogenes* the other specimen. There were no mismatches in species identified between methods. Further, multiplex-qPCR successfully identified the bacterial cause in 68/76 (89%) of culture-negative specimens.

**Table 3:** Detection of a bacterial pathogen in clinical specimens.

	Bacterial culture, n/N (%)	Multiplex-qPCR, n/N (%)	*P* value^*a*^
Any species	24/100 (24)	90/100 (90)	<0.0001
*S*. *pneumoniae*	8/100 (8)	66/100 (66)	<0.0001
*S*. *pyogenes*	14/100 (14)	20/100 (20)	ns
*H*. *influenzae*	0/100 (0)	2/100 (2)	ns
*S*. *aureus*	2/100 (2)	2/100 (2)	ns

Abbreviations: ns, not significant

^a^ *P* value refers to comparison between bacterial culture and multiplex-qPCR using M^c^Nemar test χ^2^.

### Comparison of bacterial culture and multiplex-qPCR

We used a study gold standard, defined as a positive result by either method, to compare methods (Table 4). For the 92 specimens with a positive identification by the study gold standard, the multiplex-qPCR identified a bacterial species in 90/92 (97.8%), whereas bacterial culture only identified a species in 24/92 (26.1%), McNemar’s χ^2^ 62.2, p<0.0001, equivalent to a 3.8-fold increase in species identification when using multiplex-qPCR. The most notable difference between methods was observed for the detection of *S*. *pneumoniae*, identified in eight specimens by bacterial culture and in sixty-six by multiplex-qPCR, an 8.3-fold increase, McNemar’s χ^2^ 55.1, p<0.0001.

**Table 4:** Comparison of bacterial culture and multiplex-qPCR performance against the study composite gold standard*.

	Sensitivity		NPV	
	Culture n/N (%)	qPCR n/N (%)	Culture n/N (%)	qPCR n/N (%)
Any species	24/92 (26.1)	90/92 (97.8)	8/76 (10.5)	8/10 (80)
*S*. *pneumoniae*	8/67 (11.9)	66/67 (98.5)	33/92 (35.9)	33/34 (97.1)
*S*. *pyogenes*	14/21 (66.7)	20/21 (95.2)	79/86 (91.9)	79/80 (98.8)
*H*. *influenzae*	0/2 (0)	2/2 (100)	98/100 (98.0)	98/98 (100)
*S*. *aureus*	2/2 (100)	2/2 (190)	98/98 (100)	98/98 (100)

Abbreviations: Culture, bacterial culture; qPCR, multiplex-qPCR; NPV, negative predictive value. NPV calculated as no. true negatives / no. with negative result (%). Sensitivity calculated as described for Table 1. Data are shown as percentage of samples that were positive (for either culture or qPCR) compared with the study gold standard (defined as positive by either method). Two cases that were negative by multiplex-qPCR were culture positive, one case of *S*. *pneumoniae* and one case of *S*. *pyogenes*.

### Estimating the potential clinical benefit of multiplex-qPCR

The median time-to-result for the twenty-four culture-positive specimens was 1.5 days (IQR 1–2 days). We applied a hypothetical turnaround time of 48 hours between pleural drainage and obtaining multiplex-qPCR results. In combination with culture and multiplex-qPCR sensitivity, we determined that in 61 cases, (58 culture negative and 3 culture positive), the multiplex-qPCR would have reduced the duration of untargeted therapy by a median of 20 days (IQR 17.5–23, range 1–55) compared with pleural fluid culture alone. In the 39 cases where the multiplex-qPCR would not have provided any potential clinical benefit, this was due: bacterial identity by culture becoming available within 48 hours (20 cases), multiplex-qPCR also failing to identify a bacterial cause in culture negative cases (9 cases), where identification by multiplex-qPCR would not have reduced duration (10 cases).

## Discussion

We developed a multiplex-qPCR to improve pathogen identification in paediatric empyema and facilitate rapid transition to targeted antibiotic therapy. In an observational study of hospitalized children with empyema in Melbourne, Australia, our multiplex-qPCR significantly outperformed bacterial culture, increasing pathogen identification 3.8-fold. *S*. *pneumoniae*, the predominant pathogen, was greatly underreported by bacterial culture. Children in our study received a median of 5 different antibiotics, with a median overall course duration of 25 days, highlighting the extensive use of antibiotics for this condition. We estimated that, if available, our multiplex-qPCR would reduce the duration of untargeted antibiotic therapy for children with empyema.

Our findings are consistent with other studies demonstrating that qPCR-based methods improve pathogen identification for bacterial syndromes with low culture-positive rates [18, 19]. Gosiewski et al and Yang et al demonstrated 2-fold increases over culture for blood or synovial fluid specimens respectively [20, 21]. Dunne et al. demonstrated up to a 4-fold increase when testing cerebrospinal fluid for bacterial causes of meningitis [22].

The benefit of molecular testing was most notable for *S*. *pneumoniae* in particular, where 59/67 (88%) of *S*. *pneumoniae* cases were culture negative and identified by molecular testing only. Similarly, Silva-Costa et al showed 87/98 (89%) of *S*. *pneumoniae* cases were only identified using PCR [23]. Reliance on culture-based methods for empyema diagnostics may therefore substantially underestimate *S*. *pneumoniae* as a cause. Our multiplex-PCR appeared to be less advantageous for *S*. *pyogenes*, *H*. *influenzae* and *S*. *aureus*, mainly because the numbers were small.

Our participant baseline and clinical characteristics were in line with similar Australian and international studies [24, 25]. Overall, 100 (100%) participants received antibiotic therapy prior to, or on the same day as primary pleural fluid drainage, which may have been the cause of the low numbers of positive pleural fluid cultures [8].

We assessed the potential benefit of our multiplex-qPCR for reducing the duration of untargeted antibiotic therapy, using a conservative 48 hours estimate between sample collection and assay result [26]. For assessment of the potential benefit of our multiplex-qPCR we assumed that clinicians would respond to a positive multiplex-qPCR result by prescribing targeted antibiotic therapy, even when no organism was identified by culture. We found that our multiplex-qPCR would be expected to reduce the duration of untargeted antibiotic therapy in 61% of cases by a median of 20 days. The potential clinical benefit might be greater if the multiplex-qPCR was applied with shorter turn-around times, however this will also be dependent on each laboratories capacity to perform the required upstream DNA extraction step, which adds to the duration and complexity. Automated DNA extraction systems would be an important area for future study reduce turn-around times for this process.

Our multiplex-qPCR provides a simple, sensitive and targeted approach for detecting the four bacterial species that together cause the vast majority of paediatric empyema cases globally [27, 28]. Quantitative-PCR platforms are widely available and are generally less expensive per sample compared with larger commercially available diagnostic platforms. These properties make multiplex-qPCR ideal for ad hoc testing and rapid pathogen identification, and may be of great clinical utility in settings where larger diagnostic panels may not be available or economically feasible including in low-middle income countries.

A limitation of our study is that all participants were recruited during a 48-month period and from a single site, which may limit the generalizability of our findings. Additionally, the RCH’s Department of Microbiology used cooked meat media, which may not be used in all settings for bacterial culture of pleural fluids. Encouragingly, our participant characteristics and bacterial etiology were similar to larger, multisite studies [2]. Our sample size was smaller than originally anticipated, due to reductions in empyema hospitalizations associated with COVID-19 mitigation measures [28, 29]. Of note, our assay does not detect *Mycobacterium tuberculosis*, although tuberculous empyema has a distinct clinical presentation and is usually diagnosed by stand-alone commercial assays. Being a retrospective study, our assessment of the potential clinical benefit was theoretical, and therefore has important limitation. How these results would have impacted antibiotic prescribing can only be theorised, which may limit its real-world applicability. To address this, future studies following the implementation of the multiplex-qPCR into clinical practice will enable us to determine the actual clinical impact on antibiotic prescribing and patient outcomes.

*S*. *pneumoniae* was the most common cause of empyema, identified in 67% of cases, despite 90% of participants being fully vaccinated with PCV, and 96% population coverage across Australia [30]. Given reports of serotype replacement and the emergence of serotype 3 [2, 31], it will be important to examine the pneumococcal serotypes causing empyema using molecular methods, as most *S*. *pneumoniae*-positive specimens were culture-negative in this cohort. From late 2022, Australia experienced an increase in incidence of invasive *S*. *pyogenes* disease. This was reflected in the increased proportion of empyema’s caused by *S*. *pyogenes* in 2022 and 2023 in our study. Identifying the less common causes of empyema, by unbiased methods such as 16S rDNA sequencing, metagenomics or commercial panels could inform future assay design. Additionally, the assay could be expanded to detect antibiotic resistance genes and further contribute to antimicrobial stewardship.

In conclusion, we developed a simple and robust molecular test for bacterial empyema, which greatly outperformed traditional culture-based methods and demonstrated significant potential to reduce the duration of untargeted antibiotic therapy. Our findings suggest that multiplex-qPCR is well suited for clinical diagnostics, including in low- and middle- income countries where the burden of empyema is considerable, and may benefit epidemiological studies of empyema and public health surveillance.

## Supporting information

S1 Table Species-specific targets included in the multiplex-qPCR. (DOCX)

S2 Table Bacterial isolates used to make spiked samples. (DOCX)

S3 Table Content of spiked samples used in this study. (DOCX)

S1 Fig Multiplex-qPCR performance across wide dynamic range. The multiplex-qPCR assay for empyema was assessed using a dilution series of spiked sample containing bacterial genomic DNA. The assay showed a linear dynamic range of 5x10° to 5x10^6^ genome equivalents (GE). The limit of detection was 5 GE (*lytA*, *hpd3* and *glt*) and 50 GE for *speB*. For each reaction in the multiplex-qPCR assay, the amplification efficiency ranged between 105% - 110% and a strong correlation was observed (R^2^ ≥0.998). Data are from two independent experiments run in duplicate, the error bars show the 95% confidence interval of the median.
(TIF)

S2 Fig Number of participants enrolled and bacterial aetiology. A) Between May 2019 to March 2023 100 participants were enrolled into the MATE study and pleural fluid samples collected. The numbers of eligible participants was low during 2020 and 2021, likely due to reductions in empyema hospitalizations associated with COVID-19 mitigation measures. B) *Streptococcus pneumoniae* was the predominant cause of empyema each year, while the proportion due to *S*. *pyogenes* was higher in 2022 and 2023. *Recruitment for 2019 began in May. ** Recruitment for 2023 ended in March.
(TIF)
