# Supplementary material for: Evaluation of a multiplex-qPCR for paediatric pleural empyema—An observational study in hospitalised children
Source: PLoS One. 2024 Jun 25;19(6):e0304861. doi: 10.1371/journal.pone.0304861 (PMC11198775; doi:10.1371/journal.pone.0304861)
Supplement: S1 Table — (DOCX) [file pone.0304861.s001.docx]

**S1 Table. Species-specific targets included in the multiplex-qPCR**

| Species | Target | Sequence 5’ to 3’ | Ref |
| --- | --- | --- | --- |
| *S. pneumoniae* | *lytA* | F: ACGCAATCTAGCAGATGAAGCA  R: TCGTGCGTTTTAATTCCAGCT  P: Cy5-TGCCGAAAACGCTTGATACAGGGAG-BHQ2 | (10) |
| *S. pyogenes* | *speB* | F: CTAAACCCTTCAGCTCTTGGTACTG  R: TTGATGCCTACAACAGCACTTTG  P: ROX-CGGCGCAGGCGGCTTCAAC-BHQ2 | (11) |
| *H. influenzae* | *hpd3* | F: GGTTAAATATGCCGATGGTGTTG  R: TGCATCTTTACGCACGGTGTA  P: FAM-TTGTGTACACTCCGTaGGTAAAAGAACTTGCAC-b | (12) |
| *S. aureus* | *glt* | F: CGGGTTAGGTGAATTGATTGTTTTAT  R: CGCATTTGAGCTGAAGTTG  P: Cy3TTCCATATGACCACCACGAGTCTTAGCACC-BHQ2 | (13) |

Abbreviations: F, Forward primer, R, reverse primer, P, fluorescent probe

**^a^** BHQ1, black hole quencher 1, **^b^** Spacer C3
