# Supplementary material for: Evaluation of a multiplex-qPCR for paediatric pleural empyema—An observational study in hospitalised children
Source: PLoS One. 2024 Jun 25;19(6):e0304861. doi: 10.1371/journal.pone.0304861 (PMC11198775; doi:10.1371/journal.pone.0304861)
Supplement: S2 Table — (DOCX) [file pone.0304861.s002.docx]

**S2 Table. Bacterial isolates used to make spiked samples**

| **Isolate** | **Species** | **Additional information** | **Site of isolation** |
| --- | --- | --- | --- |
| PMP1175 | *S. pneumoniae* | Serotype 19A | Invasive^a^ |
| PMP1177 | *S. pneumoniae* | Serotype 3 | Invasive^a^ |
| PMP1181 | *S. pneumoniae* | Serotype 7F | Invasive^a^ |
| PMP1345 | *S. pneumoniae* | Serotype 23B | Nasopharynx |
| PMP1384 | *S. pneumoniae* | Serotype 6C | Nasopharynx |
| PMP1421 | *S. pneumoniae* | Serotype 2 | Invasive^a^ |
| M1T1 5448 | *S. pyogenes* | M type 1 | Invasive^a^ |
| M12 611025 | *S. pyogenes* | M type 12 | Invasive^a^ |
| M75 611024 | *S. pyogenes* | M type 75 | Oropharynx |
| ATCC 10211 | *H. influenzae* | Type b | Unknown |
| Strain 1096 | *H. influenzae* | Non-typeable | Unknown |
| PMP1394 | *S. aureus* | Methicillin resistant (MRSA) | Unknown |
| PMP1395 | *S. aureus* | Methicillin sensitive (MSSA) | Unknown |

^a^ Site of isolation not known.
