# Supplementary material for: Evaluation of a multiplex-qPCR for paediatric pleural empyema—An observational study in hospitalised children
Source: PLoS One. 2024 Jun 25;19(6):e0304861. doi: 10.1371/journal.pone.0304861 (PMC11198775; doi:10.1371/journal.pone.0304861)
Supplement: S3 Table — (DOCX) [file pone.0304861.s003.docx]

**S3 Table. Content of spiked samples used in this study**

| **Species in spiked sample** | **Number of spiked samples** |
| --- | --- |
| *S. pneumoniae* | 66 |
| *S. pyogenes* | 36 |
| *H. influenzae* | 26 |
| *S. aureus* | 26 |
| *S. pneumoniae* and *S. pyogenes* | 10 |
| *S. pneumoniae* and *H. influenzae* | 6 |
| *S. pneumoniae* and *S. aureus* | 10 |
| *S. pyogenes* and *H. influenzae* | 4 |
| *S. pyogenes* and *S. aureus* | 10 |
| *H. influenzae* and *S. aureus* | 5 |
| *S. pneumoniae, S. pyogenes, H. influenzae* and *S. aureus* | 16 |
| No bacteria | 52 |
| **TOTAL** | 267 |

For samples with multiple bacterial species: *S*. *pneumoniae-* serotype 3 PMP1177*, S*. *pyogenes-* M75 611024*, H. influenzae-* Type b ATCC 10211 and *S*. *aureus-* MRSA PMP1394.
