# Supplementary figures and images for: Evaluation of a multiplex-qPCR for paediatric pleural empyema—An observational study in hospitalised children
Source: PLoS One. 2024 Jun 25;19(6):e0304861. doi: 10.1371/journal.pone.0304861 (PMC11198775; doi:10.1371/journal.pone.0304861)

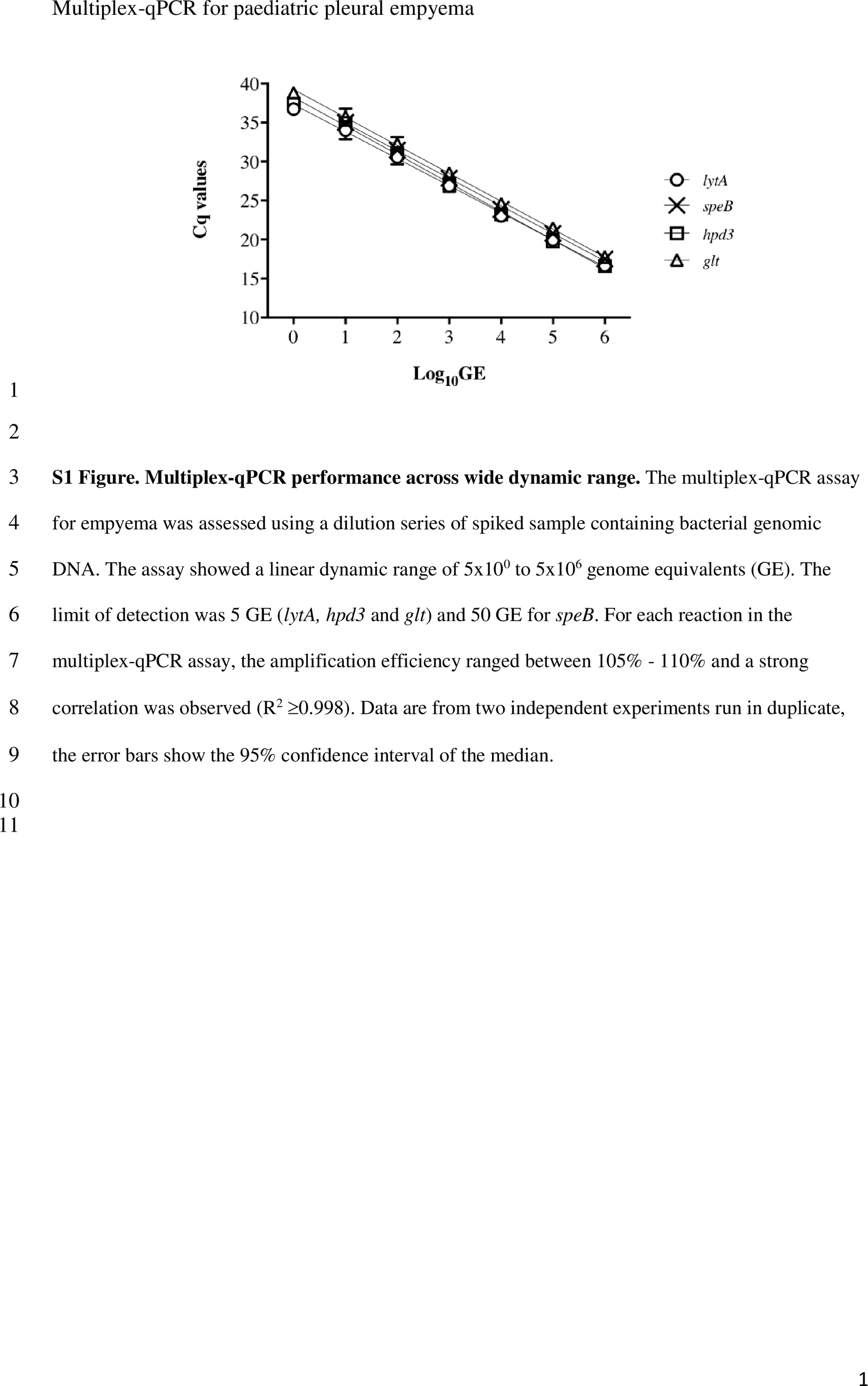

Supplement: S1 Fig — The multiplex-qPCR assay for empyema was assessed using a dilution series of spiked sample containing bacterial genomic DNA. The assay showed a linear dynamic range of 5x10° to 5x10^6^ genome equivalents (GE). The limit of detection was 5 GE (lytA, hpd3 and glt) and 50 GE for speB. For each reaction in the multiplex-qPCR assay, the amplification efficiency ranged between 105% - 110% and a strong correlation was observed (R2 ≥0.998). Data are from two independent experiments run in duplicate, the error bars show the 95% confidence interval of the median. (TIF) [file pone.0304861.s004.tif]

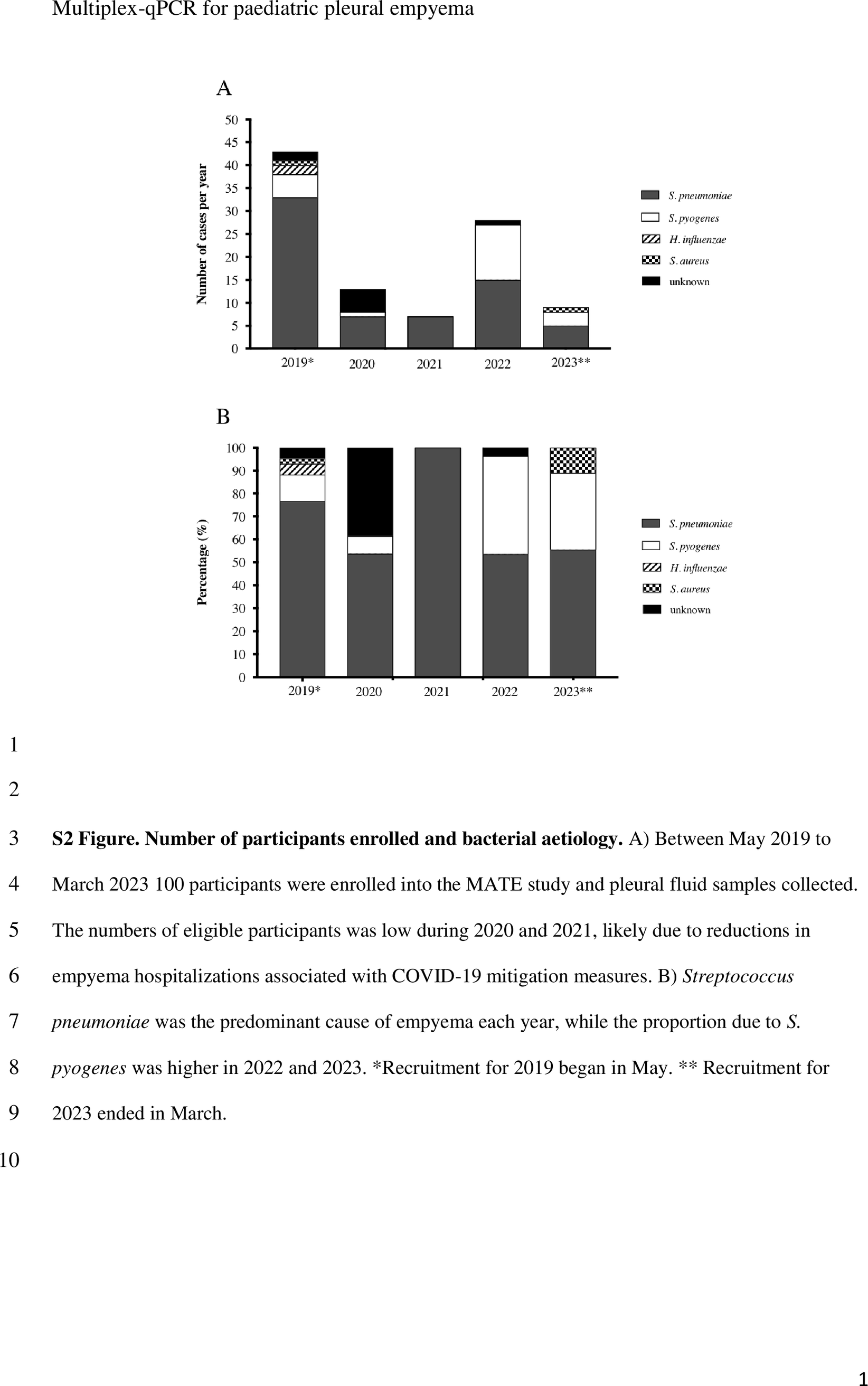

Supplement: S2 Fig — A) Between May 2019 to March 2023 100 participants were enrolled into the MATE study and pleural fluid samples collected. The numbers of eligible participants was low during 2020 and 2021, likely due to reductions in empyema hospitalizations associated with COVID-19 mitigation measures. B) Streptococcus pneumoniae was the predominant cause of empyema each year, while the proportion due to S. pyogenes was higher in 2022 and 2023. *Recruitment for 2019 began in May. ** Recruitment for 2023 ended in March. (TIF) [file pone.0304861.s005.tif]
